# The small G protein Arf6 expressed in keratinocytes by HGF stimulation is a regulator for skin wound healing

**DOI:** 10.1038/srep46649

**Published:** 2017-04-21

**Authors:** Yuki Miura, Van Ngo Thai Bich, Momoko Furuya, Hiroshi Hasegawa, Satoru Takahashi, Naohiro Katagiri, Tsunaki Hongu, Yuji Funakoshi, Norihiko Ohbayashi, Yasunori Kanaho

**Affiliations:** 1 Department of Physiological Chemistry, University of Tsukuba, 1-1-1 Tennodai, Tsukuba 305-8578, Japan; 2 Ph.D. Program in Human Biology, School of Integrative and Global Majors, University of Tsukuba, 1-1-1 Tennodai, Tsukuba 305-8578, Japan; 3 Department of Anatomy and Embryology, Faculty of Medicine and Graduate School of Comprehensive Human Sciences, University of Tsukuba, 1-1-1 Tennodai, Tsukuba 305-8578, Japan

## Abstract

The earlier step of cutaneous wound healing process, re-epithelialization of the wounded skin, is triggered by a variety of growth factors. However, molecular mechanisms through which growth factors trigger skin wound healing are less understood. Here, we demonstrate that hepatocyte growth factor (HGF)/c-Met signaling-induced expression of *the small G protein Arf6* mRNA in keratinocytes is essential for the skin wound healing. *Arf6* mRNA expression was dramatically induced in keratinocytes at the wounded skin, which was specifically suppressed by the c-Met inhibitor. Wound healing of the skin was significantly delayed in keratinocyte-specific *Arf6* conditional knockout mice. Furthermore, Arf6 deletion from keratinocytes remarkably suppressed HGF-stimulated cell migration and peripheral membrane ruffle formation, but did not affect skin morphology and proliferation/differentiation of keratinocytes. These results are consistent with the notion that Arf6 expressed in skin keratinocytes through the HGF/c-Met signaling pathway in response to skin wounding plays an important role in skin wound healing by regulating membrane dynamics-based motogenic cellular function of keratinocytes.

Cutaneous wound healing is essential to defend the body against foreign substances such as microorganisms, and a complicated biological process including three overlapping phases of inflammation, re-epithelialization, and skin remodeling. Especially, re-epithelialization of the epidermis is a key step for remodeling the primary barrier structure against invasion of microorganisms^1^,^2^. Therefore, this step should be completed immediately after wounding of the skin. In addition, the rapid and well-organized migration of keratinocytes toward the injured area of the skin is important for the re-epithelialization of the skin^3^. To support this process, a variety of growth factors such as epidermis growth factor (EGF), fibroblast growth factor (FGF) and hepatocyte growth factor (HGF) are released from fibroblasts, platelets, mesenchymal cells and keratinocytes at the wounded skin area^3^,^4^ to facilitate keratinocyte migration^5^,^6^,^7^. Accumulating evidence demonstrates the significance of these growth factors and receptors in keratinocytes migration during cutaneous wound healing. However, molecular mechanisms for cutaneous wound healing triggered by these growth factors and their receptors have not yet been well documented.

Mammalian ADP-ribosylation factor 6 (Arf6), an Arf family member of small G proteins, functions as the molecular switch by cycling between GTP-bound active and GDP-bound inactive forms^8^,^9^. In the resting state of cells, Arf6 exists as the GDP-bound inactive form, and is converted to the GTP-bound active form by the action of guanine nucleotide exchange factors upon stimulation of the cell by agonists such as hormones and growth factors. Thereafter, GTP bound to Arf6 is hydrolyzed to GDP by the GTPase activity of Arf6 under the support of GTPase-activating proteins, thereby converting Arf6 inactive^10^,^11^. Arf6 localizing to the plasma membrane and endosomes regulates membrane dynamics-based cellular events such as actin cytoskeleton reorganization^12^,^13^, membrane trafficking^14^,^15^, membrane ruffling^16^, epithelial cell migration^17^ and wound healing *in vitro*^18^ by controlling the membrane lipid composition of these organelles through lipid-metabolizing enzymes: Arf6 directly activates phosphatidylinositol 4-phosphate 5-kinase (PIP5K), which generates the versatile membrane phospholipid phosphatidylinositol 4,5-bisphosphate (PI4,5P_2_)^16^, and phospholipase D (PLD), which produces the signaling lipid phosphatidic acid (PA)^19^. In addition, it has been reported that Arf6 is activated upon cell stimulation by several growth factors, including EGF^20^ and HGF^21^,^22^. These reports led us to speculate that Arf6 plays an important role in growth factor-promoted skin wound healing.

In the present study, we investigated whether Arf6 is involved in the skin wound healing with keratinocyte-specific *Arf6* conditional knockout (K-*Arf6*-cKO) mice. Results obtained indicate that expression of *Arf6* mRNA is drastically induced in keratinocytes at the wound site after injury of the skin through the HGF/c-Met-mediated signaling to regulate the membrane dynamics-based motogenic cellular function, which is responsible for promotion of skin wound healing *in vivo*.

## Results

### Wound-dependent expression of *Arf6* mRNA in mouse skin keratinocytes

**Figure 1:**
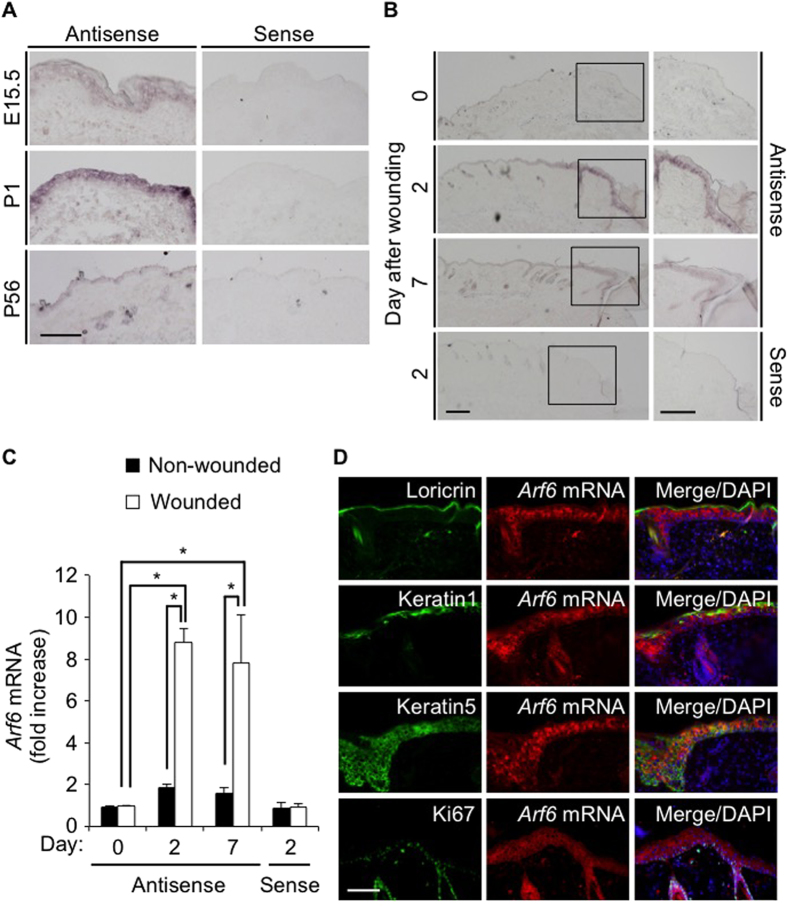
Wound-dependent expression of *Arf6* mRNA in skin keratinocytes of adult mice. (**A**) Dorsal skin sections prepared from E15.5, P1 and P56 wild type of mice were hybridized with an antisense probe1 (left panels) and a sense probe (right panels) for *Arf6* mRNA. (**B**) Dorsal skin sections prepared from 8 weeks old wild type of mice at 0, 2 and 7 days after skin wounding were hybridized as in (**A**). High magnification images of rectangular regions in the left panels are shown in the right panels. (**C**) Signal intensity of *Arf6* mRNA of wounded area relative to that of non-wounded area were analyzed and shown as mean ± SEM. Statistical significance was assessed using one-way ANOVA with Tukey’s HSD test, ^*^P < 0.05. (**D**) The injured dorsal skin sections prepared from 8 weeks old wild type of mice at 2 days after wounding were visualized by fluorescent *in situ* hybridization for *Arf6* mRNA (middle panels, red) and by immunostaining for loricrin, keratin1, keratin5, and Ki67 (left panels, green). Merged images with DAPI (blue) are shown in right panels. Scale bar, 100 μm (**A**,**D**) and 200 μm (**B**).

We have previously reported that *Arf6* mRNA is abundantly expressed in epithelial cells of various mouse tissues^23^. Consistent with this report, *Arf6* mRNA was highly expressed in the dorsal skin epidermis of embryonic day (E)15.5 and postnatal day (P)1 mice, although the expression level was extremely lower in P56 adult mice (Fig. 1A). Interestingly, the expression of *Arf6* mRNA in the skin epidermis of adult mice was dramatically enhanced when the skin was wounded: the enhancement was clearly detectable at 2 days after wounding and sustained at least up to 7 days (Fig. 1B,C), raising a possibility that Arf6 expressed in the wounded skin epidermis functions to heal skin wounds.

Skin epidermis forms the layer structures composed of basal, spinous, granular and corneum layer^24^. Fluorescence *in situ* hybridization of the wounded skin epidermis for *Arf6* mRNA, and immunostaining for loricrin, keratin1 and keratin5, which are marker proteins for granular, spinous and basal layer, respectively, revealed that *Arf6* mRNA was expressed in the keratin5-positive basal layer, but not in other layers (Fig. 1D). In addition, some population of Ki67-positive proliferating cells at the wounded site was found to express *Arf6* mRNA (Fig. 1D). These results are consistent with the notion that Arf6 is expressed in the proliferating keratinocytes in the basal layer in response to wounding, and contributes to skin wound healing.

### Deletion of *Arf6* from keratinocytes causes delay of skin wound healing

**Figure 2:**
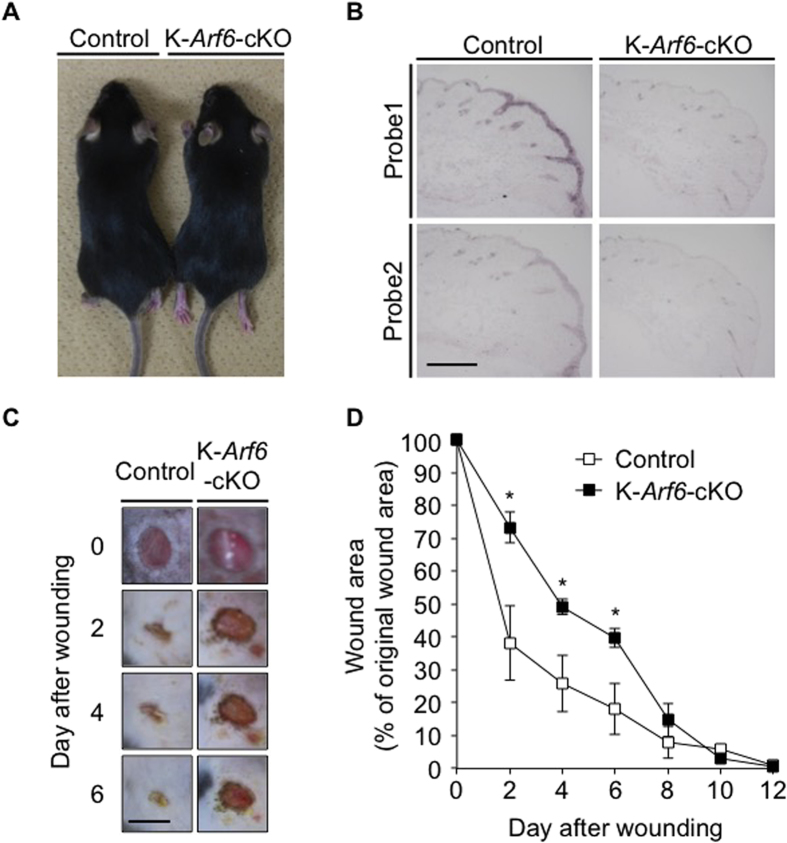
Deletion of *Arf6* from keratinocytes causes delay of skin wound healing *in vivo*. (**A**) Pictures of 9 weeks old control (left) and K-*Arf6*-cKO (right) male mice. (**B**) Knockout of *Arf6* in skin keratinocytes. Dorsal skin sections prepared from 8 weeks old control (left panels) and K-*Arf6*-cKO mice (right panels) at 2 days after wounding were hybridized with antisense probe1 and probe2 for *Arf6* mRNA. (**C**,**D**) *In vivo* skin wound healing. Representative images of the time-dependent dorsal skin wound healing in 8 weeks old control (left panels) and K-*Arf6*-cKO (right panels) mice (**C**) and quantitative data for wound area (**D**). Data shown in (**D**) are means ± SEM from 4 independent experiments. Statistical significance was assessed using Student’s *t*-test, ^*^P < 0.05. Scale bar, 300 μm (**B**) and 5 mm (**C**).

To address the notion described above that Arf6 expressed in skin keratinocytes in response to wounding is involved in skin wound healing, we generated conditional knockout mice specifically lacking *Arf6* in skin keratinocytes (K-*Arf6*-cKO: *K14-Cre*; *Arf6*^*flox/flox*^) and analyzed skin wound healing in these mice. K-*Arf6*-cKO mice were born at the expected Mendelian ratio, overtly healthy and able to reproduce (data not shown) without any obvious abnormalities in their skin and hair growth (Fig. 2A). Expression of *Arf6* mRNA induced by skin wounding, which was observed in wild type of mice, was almost completely suppressed in K-*Arf*6-cKO mice (Fig. 2B), demonstrating the successful deletion of *Arf6* from skin keratinocytes. In K-*Arf*6-cKO mice, as expected, closure of a full-thickness wound was delayed compared with control mice (Fig. 2C,D), supporting the notion described above.

### HGF/c-Met signaling promotes the expression of *Arf6* mRNA in the wounded skin

**Figure 3:**
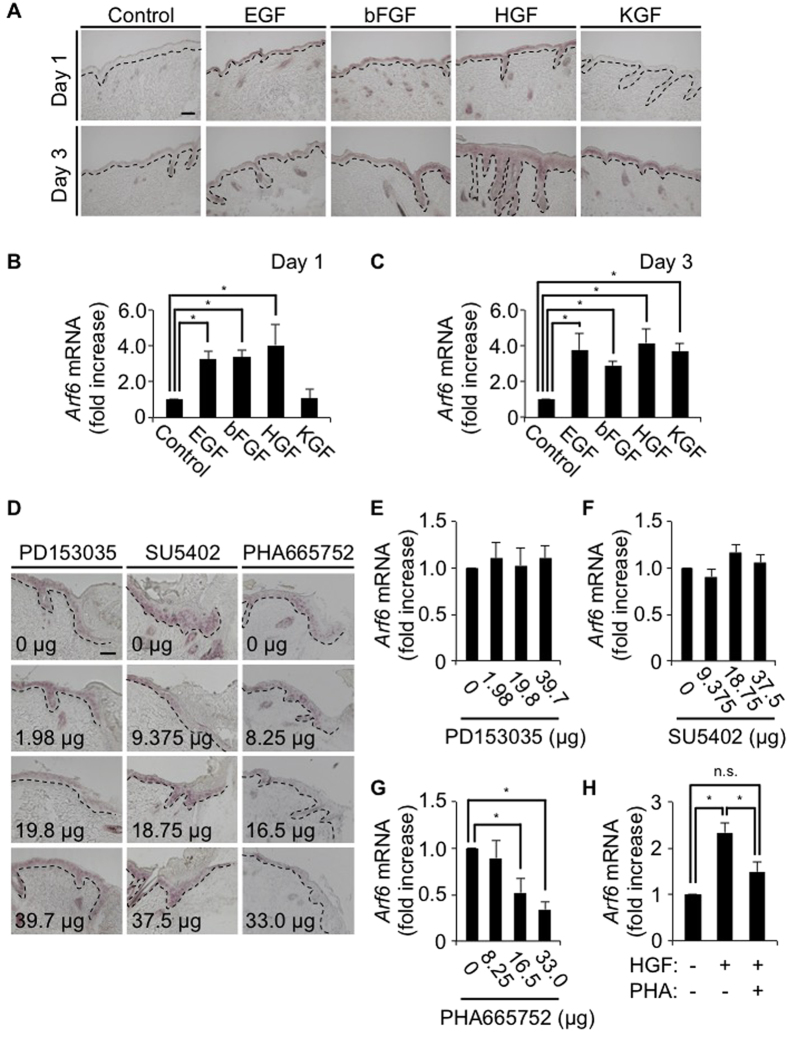
HGF/c-Met signaling promotes the expression of *Arf6* mRNA in the wounded skin. (**A**) Eight weeks old wild type of mice were treated without (control) or with EGF, bFGF, HGF or KGF. After 1 day (upper panels) and 3 days (lower panels) of treatment, dorsal skin sections were prepared and hybridized with an antisense probe1 for *Arf6* mRNA. (**B**,**C**) Signal intensity of *Arf6* mRNA at 1 day (**B**) and 3 days (**C**) after treatment were measured. (**D**–**G**) Dorsal skins of 8 weeks old wild type mice were wounded and treated with inhibitors specific to EGFR, FGFR and c-Met (PD153035, SU5402 and PHA665752, respectively) at the indicated doses for 2 days, and hybridized with an antisense probe1 for *Arf6* mRNA (**D**). Signal intensity of *Arf6* mRNA at the wounded skin treated without or with PD153035 (**E**), SU5402 (**F**) and PHA665752 (**G**) were measured. (**H**) *Arf6* mRNA levels in primary cultured keratinocytes treated without or with 50 ng/ml of HGF in the presence or absence of 0.5 μM of PHA665752 were analyzed by qPCR. Data show the means ± SEM from three (**B–G**) and five independent experiments (**H**). Statistical significance was assessed using one-way ANOVA with Dunnett’s multiple comparison test (**B**,**C**,**E**,**F**,**G**) and Tukey’s HSD test (**H**); ^*^P < 0.05, n.s., not significant. Scale bar, 100 μm. Dotted lines delineate the border between epidermis and dermis.

Another issue to be clarified is which signaling induces *Arf6* mRNA expression in response to skin wounding. It has been reported that epidermal growth factor (EGF), basic fibroblast growth factor (bFGF/FGF2), hepatocyte growth factor (HGF) and keratinocyte growth factor (KGF/FGF7) are released from fibroblasts, mesenchymal cells and keratinocytes at the wounded skin area, and involved in wound healing^3^,^4^. Administration of these growth factors into the skin of wild type mice elicited the expression of *Arf6* mRNA in the skin epidermis after 1-3 days of administration (Fig. 3A–C), indicating that these growth factors have a potential to promote the *Arf6* mRNA expression in the skin. To identify growth factors and their receptors physiologically responsible for the induction of *Arf6* mRNA expression in the wounded skin, specific inhibitors, PD153035 for the EGF receptor^25^, SU5402 for the bFGF and KGF receptors^26^, and PHA665752 for the HGF receptor c-Met^27^, were administered to the wounded skin, and the expression of *Arf6* mRNA was analyzed by *in situ* hybridization. The *Arf6* mRNA expression induced by wounding was specifically suppressed by the c-Met inhibitor, but not by other inhibitors for EGF, bFGF and KGF receptors, in a dose-dependent manner (Fig. 3D–G). Moreover, the treatment with HGF up-regulated *Arf6* mRNA expression in primary cultured keratinocytes, and this up-regulation was suppressed by the c-Met inhibitor PHA665752 (Fig. 3H). These results suggest that HGF/c-Met signaling acts as a physiological trigger of *Arf6* mRNA expression in keratinocytes at the injured skin to promote wound healing.

**Figure 4:**
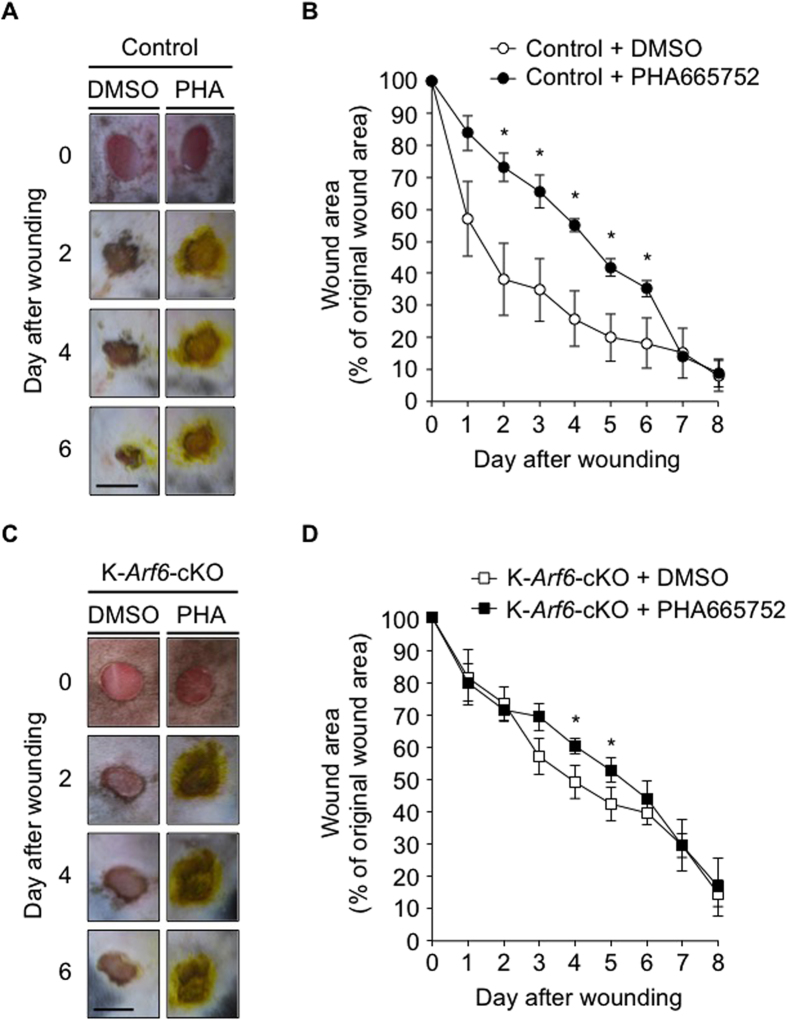
Effects of c-Met inhibitor on wound healing in control and K-*Arf6*-cKO mice. (**A–D**) DMSO or 33 μg of PHA665752 were applied every day after wounding, and wound closure was analyzed up to 8 days. Representative images of the dorsal skin wound healing in control (A) and K-*Arf6*-cKO mice (C). Quantitative data for control (**B**) and K-*Arf6*-cKO (**D**) mice were shown as means ± SEM from four independent experiments. Statistical significance was assessed using Student’s *t*-test, ^*^P < 0.05. Scale bar, 5 mm.

In support of this notion, the treatment of control mice with the c-Met inhibitor PHA665752 significantly delayed wound healing (Fig. 4A,B). However, it was found that the c-Met inhibitor still showed the small suppression of skin wound healing in K-*Arf6*-cKO mice (Fig. 4C,D). These results indicate that other factor(s) is also involved in the HGF/c-Met axis-induced signaling pathway of skin wound healing, although Arf6 at least in part functions as a downstream molecule in this signaling pathway. Another possibility is that other Arf isoforms are up-regulated by knockout of *Arf6* or stimulation with HGF to compensate for the loss of Arf6. This is unlikely because expression levels of *Arf1* and *Arf5* mRNAs in primary cultured keratinocytes were not affected by *Arf6* knockout and HGF stimulation, while *Arf6* mRNA expression levels in control and HGF-stimulated cells were significantly decreased by the deletion of Arf6 (Supplementary Fig. S1).

### HGF-stimulated cell migration and peripheral membrane ruffle formation are impaired in *Arf6*-deleted keratinocytes

**Figure 5:**
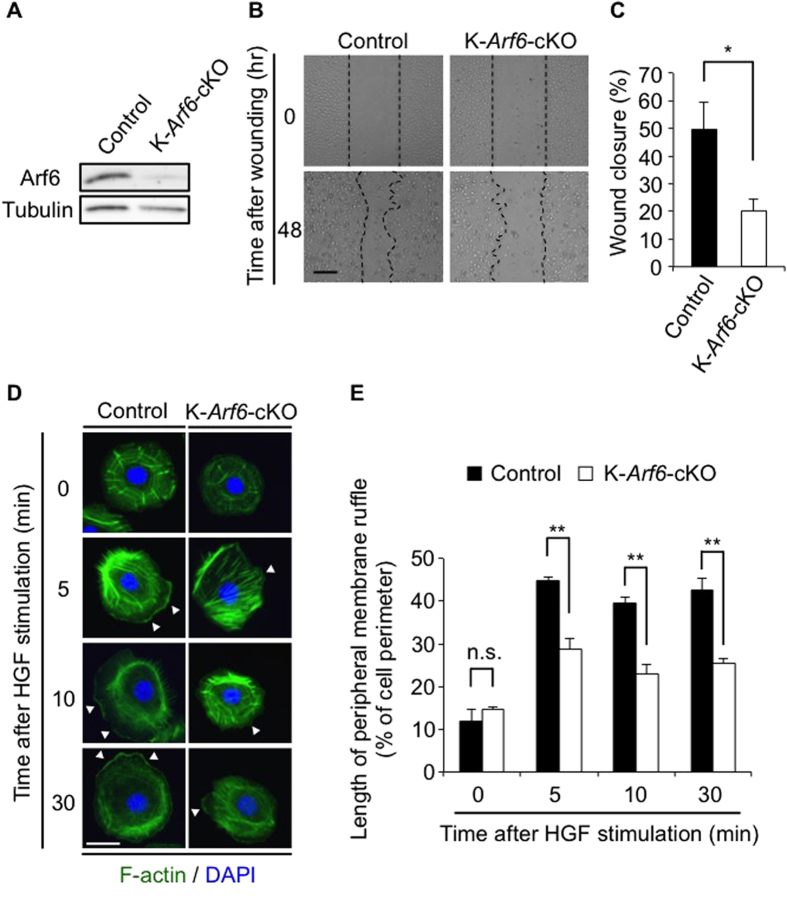
HGF-stimulated cell migration and peripheral membrane ruffle formation are impaired in *Arf6*-deleted keratinocytes. (**A**) Ablation of *Arf6* from keratinocytes. Lysates of primary cultured keratinocytes prepared from control and K-*Arf6*-cKO mice were immunoblotted with anti-Arf6 and anti-tubulin antibodies. (**B**,**C**) Scratch-wound assay of primary cultured keratinocytes treated with 50 ng/ml of HGF. Representative images (**B**) and quantitative data of wound closure at 48 hr after scratching (**C**) were shown. (**D**,**E**) Primary cultured keratinocytes prepared from control and K-*Arf6*-cKO mice were stimulated with 50 ng/ml of HGF for the indicated time, and immunostained with F-actin (green) and DAPI (blue). Shown are representative images (**D**) and quantitative data of the peripheral membrane ruffle formation (percentage of peripheral membrane ruffle length to cell perimeter) of 50 cells (**E**) from three independent experiments. Allow heads indicate the region of peripheral membrane ruffles. Data show the mean ± SEM of five (**C**) and three independent experiments (**E**). Statistical significance was calculated using Student’s *t*-test; n.s., not significant, ^*^P < 0.05, ^**^P < 0.01, Scale bar, 200 μm (**B**), 10 μm (**D**).

Migration of keratinocytes is a critical cell event for skin wound healing. It has been reported that Arf6 plays essential roles in migration of epithelial cells *in vitro*^17^,^18^. These reports, taken together with the result shown above that HGF/c-Met signaling is responsible for the expression of *Arf6* mRNA in keratinocytes, led us to speculate that delay of skin wound healing in K-*Arf*6-cKO mice might be attributable to the impairment in cell migration of keratinocytes. To address this assumption, wound closure was measured by the *in vitro* scratch-wound healing assay with primary cultured keratinocytes prepared from control and K-*Arf*6-cKO newborn mice. The expression level of Arf6 was significantly diminished in primary cultured keratinocytes prepared from K-*Arf*6-cKO mice (Fig. 5A), demonstrating that *Arf6* is successfully, but not perfectly, knocked-out. In *Arf*6-deficent keratinocytes, wound closure stimulated by HGF remarkably delayed (Fig. 5B,C). Furthermore, it was found that HGF-stimulated peripheral membrane ruffling formation, which is essential for cell migration, was markedly suppressed in *Arf*6-deficient keratinocytes (Fig. 5D,E). These results strongly support the notion that delay of skin wound healing in K-*Arf*6-cKO mice is attributed to the defect in the peripheral membrane ruffling formation and subsequent cell migration of keratinocytes.

### Skin morphology and proliferation/differentiation of keratinocytes during skin wound healing were not affected by *Arf6* deletion

**Figure 6:**
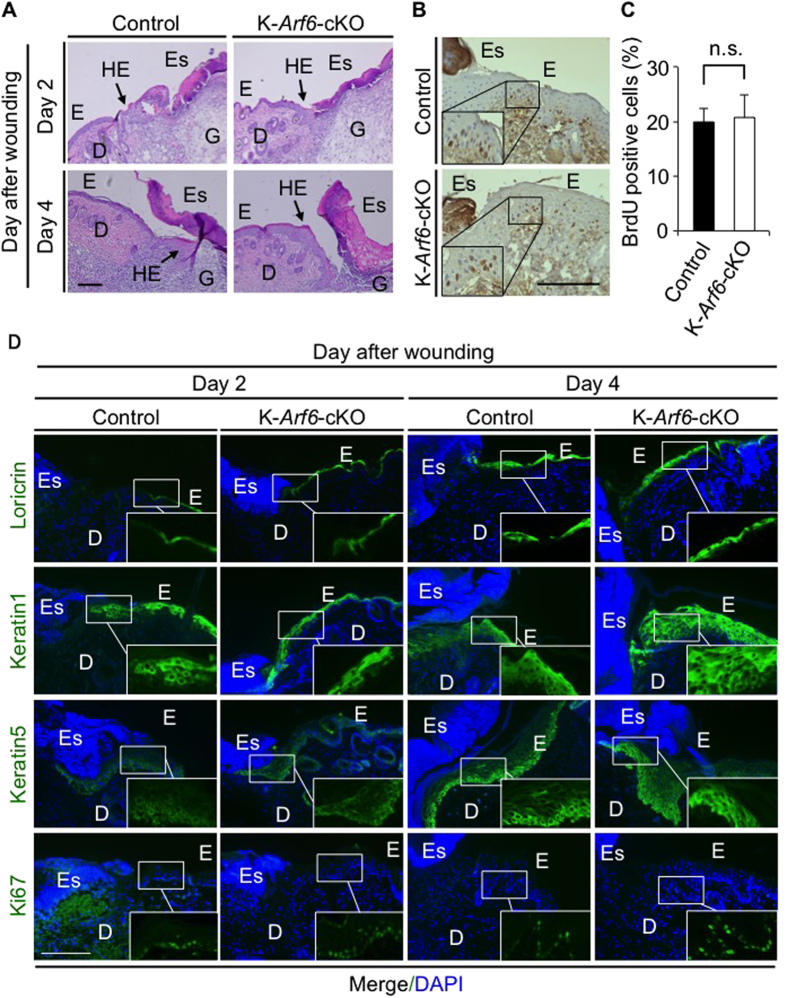
Skin morphology and proliferation/differentiation of keratinocytes during skin wound healing were not affected by *Arf6* deletion. (**A**) H&E staining of the wounded dorsal skins of control (left panels) and K-*Arf6*-cKO mice (right panels). After 2 and 4 days of skin wounding, skin sections were prepared and stained with H & E. E, epidermis; D, dermis; Es, eschar; G, granulation tissue, HE; hyperproliferative epithelium. (**B,C**) Proliferation of keratinocytes at the wounded dorsal skin. Dorsal skins of control and K-*Arf6*-cKO mice were wounded, and injected with BrdU after 3 days of wounding. After 2 hr of BrdU injection, the dorsal skin sections were prepared, immunostained with anti-BrdU antibody (brown) and counterstained with haematoxylin (violet) (**B**). Number of BrdU-positive cells at wounded skins shown by rectangle in (**B**) were counted (**C**). (**D**) Structure of wounded dorsal skins of control and K-*Arf6*-cKO mice. After 2 and 4 days of dorsal skin wounding, skin sections were prepared and immunostained for loricrin, keratin1, keratin5 and Ki67 (green). Nuclei were also stained with DAPI (blue). Data shown in (**C**) are means ± SEM for three independent experiments. Statistical significance was assessed by Student’s *t*-test; n.s., not significant. Scale bar, 200 μm.

Other cellular events essential for skin wound healing are proliferation and differentiation of keratinocytes at the wounded skin. Formation of hyperproliferative epithelium and eschar at the wounded site were similarly observed in control and K-*Arf*6-cKO mice as analyzed by haematoxylin-eosin (H & E) staining (Fig. 6A). The number of proliferating keratinocytes at the wounded site, which was assessed by the BrdU staining of keratinocytes, was comparable between control and K-*Arf*6-cKO mice (Fig. 6B,C). Furthermore, the layer structure of the epidermis visualized by immunohistochemistry for loricrin, keratin1 and keratin5, and number of Ki67-positive cells were almost the same between control and K-*Arf6*-cKO mice (Fig. 6D). Thus, layer structure of the epidermis and proliferation/differentiation of keratinocytes at the wound site are not affected by deletion of *Arf6*, leading to the conclusion that delay of skin wound healing in K-*Arf*6-cKO mice is attributed to the defect in cell migration of keratinocytes caused by the impaired ruffle formation.

## Discussion

The results obtained in this study provide evidence for the first time that expression of *Arf6* mRNA in keratinocytes is induced via HGF/c-Met signaling when the skin is wounded, and thus upregulated Arf6 at least in part plays an important role in skin wound healing by regulating the membrane dynamics-based motogenic function of keratinocytes.

It has been reported that expression of *HGF* and *c-Met* mRNAs is elicited in keratinocytes at wound edge when the skin is injured^7^, although the HGF protein is expressed only in hair follicle mesenchyme of normal skin, but not in epidermis, and c-Met exists in neighboring hair bulb keratinocytes^28^. Similar to *HGF* and *c-Met* mRNA expression, expression of *Arf6* mRNA in keratinocyte was induced in response to skin wound (Fig. 1B–D), which was specifically inhibited by the c-Met inhibitor PHA665752 in a dose-dependent manner (Fig. 3D–G), suggesting that HGF/c-Met axis is a key signaling pathway for the induction of *Arf6* mRNA expression. Although the molecular mechanism(s) through which the HGF/c-Met signaling induces *Arf6* mRNA expression remains to be clarified, ERK/MAPK, PI3K and the transcription factor SP1 may coordinately play roles in the HGF/c-Met-induced *Arf6* mRNA expression. This idea is consistent with the report showing that SP1 is required for the EGF-induced expression of *Arf6* mRNA in the human glioblastoma cell line U87 cells^29^. The idea is further supported by the report that HGF treatment of the human keratinocyte cell line HaCaT cells increases the phosphorylation of SP1, which enhances its transcription activity, through the regulation of MAPK and PI3K^30^. Thus, it is reasonable to speculate that ERK/MAPK, PI3K and SP1 coordinately mediate the induction of *Arf6* mRNA expression through HGF/c-Met axis in keratinocytes at the wounded skin. Clarification of the molecular mechanisms for HGF-dependent induction of *Arf6* mRNA expression in response to skin wound could contribute to developing novel treatment protocols and drugs to enhance skin wound healing.

Although we provided evidence that Arf6 at lease in part plays an important role in wound healing of the skin *in vivo*, we cannot totally rule out the possibility that other factor(s) is also involved in the skin wound healing promoted by HGF. This possibility is raised by the observation that the effect of *Arf6* knockout on skin wound healing was smaller than that of the c-Met inhibitor (Figs 2 and 4) and that delay of the wound healing was still observed in K-*Arf6*-cKO mice (Fig. 4C,D). It has been reported that HGF/c-Met axis plays crucial roles in wound healing by regulating keratinocyte migration and proliferation^7^. The results obtained in this study demonstrate that the function of Arf6 is limited to the HGF-stimulated keratinocyte migration: Deletion of Arf6 from keratinocytes inhibited the HGF-dependent keratinocyte migration (Fig. 5B,C), but not proliferation (Fig. 6B,C). From these observations, it is plausible that Arf6 plays an important role in the motogenic cellular events of keratinocytes and other factor(s) is essential for keratinocyte proliferation in the HGF/c-Met signaling pathway of cutaneous wound healing.

Another issue to be defined is the molecular mechanisms by which Arf6 regulates the HGF-dependent membrane ruffle formation which is required for cell migration of keratinocytes. Although further experiments are required to clarify this issue, PIP5K is easily speculated to be a downstream effector of Arf6 in the signaling pathway of HGF-induced membrane ruffle formation of keratinocytes, based on the following reports: (1) PIP5K functions as a downstream effector of Arf6 in the signaling pathway for EGF-stimulated membrane ruffle formation in HeLa cells^16^; (2) Arf6 is activated in hepatocytes and vascular endothelial cells upon HGF stimulation^21^,^22^; (3) HGF stimulation of the human hepatocyte carcinoma cell line HepG2 cells activates PIP5K through Arf6 (unpublished data); (4) the versatile membrane phospholipid PI4,5P_2_ produced by PIP5K reorganizes actin cytoskeleton at the plasma membrane^16^, which is a critical cellular events for cell migration^31^. PI4,5P_2_ at the plasma membrane interacts with actin binding proteins such as gelsolin^32^ and cofilin^33^ to reorganize actin filament which is essential for membrane ruffle formation to promote cell migration. In addition, PI4,5P_2_ is an essential recruiter/activator of focal adhesion (FA) components such as tailin^34^, ERM proteins^35^, and vinculin^36^ to regulate the cell adhesion to extracellular matrix which is also essential for cell migration. Therefore, PI4,5P_2_ generated at the plasma membrane by Arf6-PIP5K axis would facilitate the membrane ruffle formation and cell adhesion at the leading edge of the cell to promote keratinocyte migration during wound healing of the skin.

Alternative downstream molecule of Arf6 is the lipid metabolizing enzyme PLD1. Arf6 has been reported to directly activate PLD1^19^, and activated PLD1 plays an important role in the migration of epithelial cells^18^. Furthermore, we have previously demonstrated that PLD1 is essential for cytokine-induced migration of neutrophils by controlling actin cytoskeleton reorganization^37^. Thus, Arf6/PLD1 axis might be critical for the regulation of actin cytoskeleton reorganization-based motogenic function of keratinocytes during wound healing of the skin.

Trafficking of the adhesion molecule β1 integrin between the plasma membrane and endosomes^38^, which regulates attachment/detachment of cells to/from extracellular matrix^39^, is a crucial cellular event for Arf6-mediated peripheral membrane ruffling^40^ and cell migration of keratinocytes^41^. Since production of PI4,5P_2_ at the plasma membrane and the recycling endosome has been implicated in the β1 integrin trafficking, including its internalization^42^ and/or recycling^43^, it is plausible that the Arf6/PIP5K axis-dependent PI4,5P_2_ production at these cellular compartments regulates membrane ruffle formation and cell migration of keratinocytes through β1 integrin trafficking. This idea is further supported by the reports demonstrating that Arf6 is involved in β1 integrin internalization to disassemble FA during keratinocytes migration^44^ and in HGF-induced β1 integrin recycling in vascular endothelial cells to form FA and promote cell migration^22^. Thus, Arf6 could regulate cell migration by regulating multiple steps of β1 integrin trafficking process.

In the present study, we provide insight into the physiological significance of HGF/c-Met/Arf6 signaling in the skin wound healing. In addition to the skin, other tissues, such as liver, lung and kidney, express Arf6^23^,^45^, suggesting that Arf6 plays an key role in wound healing-related pathophysiological phenomena in these tissues. In support of this assumption, cytohesin family members of Arf6 GEFs, which regulate Arf6 activity, are required for the HGF/c-Met signaling-dependent recovery of acute kidney injury^46^. Thus, it is of interesting to investigate pathophysiological roles of HGF/c-Met/Arf6 signaling in a wide variety of tissues.

## Methods

### Animals and generation of K-*Arf6*-cKO mice

All experiments with mice were carried out according to the Guideline for Proper Conduct of Animal Experiments, Science Council of Japan. The protocols for experiments with mice were approved by Animal Care and Use Committee, University of Tsukuba.

C57BL/6J mice were purchased from SLC, Japan. K-*Arf6*-cKO mice were generated by mating *Arf6*^*flox/flox*^ mice^22^, which were used as control for K-*Arf6*-cKO mice, with *K14-Cre* mice which express *Cre* after E15^47^. Age- and sex-matched 8 to 12 weeks old mice were used for *in vivo* experiments.

### Analyses for effects of growth factors and their receptor inhibitors on *Arf6* mRNA expression in the skin

To assess the effects of growth factors on *Arf6* mRNA expression in the normal skin, 300 ng of EGF (Roche), 140 ng of bFGF (Pepro Tech), 120 ng of HGF (Sigma-Aldrich) or 120 ng of KGF (Pepro Tech) in 200 μl of growth factor-reduced matrigel matrix (BD Biosciences) was subcutaneously injected into the dorsal skin of adult mice, and skin sections were prepared at 1 or 3 days after injection.

To investigate the effects of inhibitors for growth factor receptors on the expression of *Arf*6 mRNA in the wounded skin of adult mice, inhibitors of EGFR (PD153035, TOCRIS), FGFR (SU5402, SANTA CRUZ) and c-MET (PHA665752, SANTA CRUZ) at the indicated dose were topically applied to the wounded skin every day after wounding, and skin sections were prepared 2 days after wounding for *in situ* hybridization of *Arf6* mRNA.

### *In situ* hybridization

*In situ* hybridization for *Arf6* was performed with antisense (probe1 and probe2) and sense cRNA probes for mouse *Arf6* as described previously^23^. Antisense probe 1 and sense probe were prepared as previously reported^23^. A fragment of *Arf6* DNA template for antisense probe2 was amplified with PCR primers, 5′-AATGAGCGTCCTCCACCCAG-3′ and 5′-GCGTAATACGACTCACTCTATAGGGCGACCTGACATTACCTGTCTGACA-3′, then probe2 was produced as previously reported^23^. Mice were anesthetized with 20 ml/kg body weight of Avertin, and intracardially perfused with 4% paraformaldehyde (PFA)/phosphate-buffered saline (PBS). Dorsal skin were dissected, post-fixed with 4% PFA/PBS at 4 °C overnight, transferred into 30% sucrose/PBS, incubated at 4 °C overnight, and embedded into OCT compound (Sakura Finetek). Skin sections were prepared using cryostat (Leica Microsystems), fixed with 4% PFA/PBS at room temperature (r.t.) for 10 min, and washed three times with PBS. After being immersed in 0.1 M triethanolamine containing 0.25% acetic anhydride for 10 min, sections were washed with PBS three times, then incubated in the prehybridization buffer (50% formamide, 5 x SSC (750 mM NaCl, 75 mM sodium citrate, pH 7.5), 1 × Denhardt’s, 250 μg/ml of tRNA, and 500 μg/ml of herring sperm DNA) at 4 °C overnight. Skin sections were then hybridized with cRNA probes in the hybridization buffer (50% formamide, 300 mM NaCl, 20 mM Tris-HCl, pH 8.0, 5 mM EDTA, 10 mM Na_2_HPO_4,_ 10% dextran sulfate, 1 × Denhardt’s, 500 μg/ml tRNA, and 200 μg/ml herring sperm DNA) at 65 °C overnight. The sections were washed with 5 × SSC for 10 min and with 0.2 x SSC (30 mM NaCl, 3 mM sodium citrate, pH 7.5) at 65 °C for 30 min four times, subsequently rinsed with 0.2 × SSC and with buffer A (0.1 M Tris-HCl, pH 7.5, 0.15 M NaCl) at r.t. for 5 min, and blocked with 10% normal sheep serum (Millipore) in buffer A at r.t. for 1 hr. The sections were reacted with the alkaline phosphatase-conjugated anti-DIG antibody (11 093 274 910, Roche) in buffer A supplemented with 1% sheep serum at 4 °C for overnight, washed three times with 0.1% Triton X-100/buffer A for 30 min, and incubated in buffer consisting of 0.1 M Tris-HCl, pH 9.5, 0.1 M NaCl, and 50 mM MgCl_2_ at r.t. for 5 min. The sections were then developed with nitro blue tetrazolium/5-bromo-4-chloro-3-indolyl-phosphate (NBT/BCIP) (Roche). Images were obtained with Biozero BZ-8000 or BZ-X710 microscope (Keyence), and signal intensity of *Arf6* mRNA was measured with ImageJ (NIH).

### Fluorescence *in situ* hybridization

After sections were blocked as described in “*In situ* hybridization”, *Arf6* mRNA in the section was sequentially stained with alkaline phosphatase-conjugated anti-DIG antibody and with Fast Red (11 496 549 001, Roche)/0.1 M Tris-HCl, pH 8.0. After fluorescence *in situ* hybridization for *Arf6* mRNA, sections were also immunohistochemically stained with first antibodies specific to loricrin (PRB-145P, Covance), keratin1 (PRB-165P, Covance), keratin5 (PRB-160P, Covance), and Ki67 (ab15580, abcam) and with the second antibody Alexa Fluor^®^ 488 goat anti-rabbit IgG antibody (Life Technologies) (Fig. 1). Finally, the sections were counterstained with 4′, 6-diamidino-2-phenylindole (DAPI, Molecular probe)/PBS. Images were obtained with Biozero BZ-8000 (Keyence).

### *In vivo* skin wound healing assay

Full-thickness wound was generated on dorsal skin of control and K-*Arf6*-cKO mice using 4.5 mm leather punch (BIGMAN), and wound closure of dorsal skin was analyzed up to 12 days after the wounding. To examine the effects of the c-Met inhibitor PHA665752 on the skin wound healing in control and K-*Arf6*-cKO mice, mice anesthetized with isoflurane (Intervet, Inc.) were applied with 33 μg of PHA665752 topically at the wounded skin area every day after wounding, and wound closure was analyzed up to 8 days after wounding. Digital images of the wounded skin were obtained by LUMIX DMC-L10 camera (Panasonic). Wound area was measured, and the percentage of wounded area was calculated with ImageJ (NIH).

### Histological analysis and immunohistochemistry of the skin

H & E staining of the mouse skin were carried out according to the standard method^48^. Images were obtained with Biozero BZ-8000 microscope (Keyence).

For the immunohistochemical analysis of the skin shown in Fig. 6, skin sections were washed three times with PBS, and blocked with H-PHT (1.5% heat-inactivated goat serum in 0.1% Triton X-100/PBS) for 1 hr. Sections were then incubated with anti-loricrin, anti-keratin1, anti-keratin5, or anti-Ki67 antibody at 4 °C overnight. After washing three times with PBST (0.1% Tween-20/PBS), sections were incubated with Alexa Fluor^®^ 488 goat anti-rabbit IgG antibody at r.t. for 1 hr,  and counterstained with DAPI. Images were obtained with Zeiss Axio Observer Z1 equipped with an AxioCam MRm (ZEISS).

### Assay for proliferation of skin keratinocytes

Proliferation of skin keratinocytes *in vivo* was assessed using BrdU (nakalai tesque). Mice were wounded on their dorsal skin and intraperitoneally administrated with 250 mg/kg body weight of BrdU at 3 days after wounding. After 2 hr of BrdU administration, sections of the wounded skin were prepared as described above in “*In situ* hybridization”. After sections were washed three times with PBS, endogenous peroxidase in the sections was inactivated with 3% H_2_O_2_/methanol solution at r.t. for 15 min, and subsequently antigens in the section was retrieved by heating the section in 0.01 M sodium citrate, pH 6.0, with microwave oven. The sections were washed three times with 0.1% Triton X-100/PBS, blocked with H-PHT at r.t. for 1 hr, then incubated with anti-BrdU antibody (B2531, Sigma-Aldrich) in H-PHT at 4 °C overnight. After washing three times with PBST, sections were incubated with biotinylated anti-mouse/anti-rabbit IgG antibody (BA-1400, VECTOR) in H-PHT at r.t. for 1 hr. The sections were washed three times with PBST, and incubated with ABC Reagent kit (PK-6100, VECTOR) at r.t. for 30 min. After being washed twice with PBS, sections were reacted with DAB (DAKO). Sections were also counterstained with haematoxylin for nuclear staining. Images were obtained by BZ-X710 microscope (Keyence). The number of BrdU-positive cells at wound site were measured by ImageJ (NIH).

### Primary culture of keratinocytes

The skin obtained from dorsal and ventral region of control and K-*Arf6*-cKO newborn mice were placed on the filter paper filled with 0.25% trypsin/PBS at 4 °C overnight. Epidermis were separated from dermis, and collected in EMEM.06 (Eagle’s essential medium containing 0.06 mM CaCl_2_). After gentle shaking, cells were harvested on collagen type IV-coated dishes in keratinocyte growth medium consisting of primary fibroblast conditioned medium, low-calcium medium [EMEM.06 containing 10% chelexed fetal bovine serum], 2 ng/mL EGF (BD biosciences), 0.75 mM Aminoguanidine nitrate (Sigma-Aldrich), 10^-10^ M Cholera toxin (Sigma-Aldrich), and 0.4 μg/mL Hydrocortisone (Sigma-Aldrich), as described previously^49^. These primary cultured keratinocytes were subjected to assays for *in vitro* wound-scratch, membrane ruffle formation and real-time PCR.

### *In vitro* scratch-wound assay

Keratinocytes prepared as described above were starved in EMEM.06 (starved medium) for 16 hr. After medium was replaced to and cultured in the starved medium containing 10 μg/ml of Mitomycin C (Sigma-Aldrich) to prevent cell proliferation^50^, a single wound line was made with scraping cells with 0.2 ml yellow pipette tip. After washing out the detached cells with PBS twice, keratinocytes were stimulated with 50 ng/ml of HGF (a kind gift from Dr. K. Miyazawa, University of Yamanashi) for 48 hr. Images of the cell culture at 0 and 48 hr after scratching were obtained by Biozero BZ-8000 microscope (Keyence). Wound closure was measured by BZ analyzer (Keyence).

### Peripheral membrane ruffle formation

Primary cultured keratinocytes prepared from control and K-*Arf6*-cKO mice as described above were starved for 18 hr, and stimulated with 50 ng/ml of HGF for the indicated times. Cells were then fixed with 4% PFA/PBS at r.t. for 30 min, and stained with Alexa Fluor^®^ 488 phalloidin (Life Technologies) and DAPI (Molecular probe). Fluorescence images were acquired with Zeiss Axio Observer Z1 equipped with an AxioCam MRm (ZEISS). Length of membrane ruffle and cell periphery length in single cell were measured using ImageJ (NIH), and percentage of membrane ruffle length to cell periphery length was calculated.

### Real-time PCR

For the analysis of *Arfs* mRNA expression in primary cultured keratinocytes, keratinocytes prepared as described above were starved for 18 hr, and stimulated with 50 ng/ml of HGF for 3 hr in the presence or absence of 0.5 μM of PHA66575. Total RNA was extracted from the cell using Trizol reagent (Life Technologies) according to manufacturer’s protocol. The obtained RNA was reverse-transcribed using SuperScript III Reverse Transcriptase (Life Technologies), and real-time PCR was performed with THUNDERBIRD SYBR qPCR mix (TOYOBO) and Applied Biosystems 7500/7500 Fast Real-Time PCR Systems (Thermo Fisher Scientific). *Actb* (β-actin) was used as an internal control. The sequences of primers were as follows: *Arf1*, 5′-ATGCGCATTCTCATGGTG-3′ and 5′-AACAGTCTCCACATTGAAACCA-3′; *Arf5*, 5′-AGTCTGCTGATGAACTCCAGAA-3′ and 5′-GCTTGTTGGCAAACACCA-3′; *Arf6*, 5′-TGCCTAAACTGGAGGAAACTTGAA-3′ and 5′-ACCACATCTCACCTGCAACATT-3′; *Actb*, 5′-GATCATTGCTCCTCCTGAGC-3′ and 5′-GTCATAGTCCGCCTAGAAGCAT-3′.

### Western blotting for Arf6 protein

Protein levels of Arf6 in keratinocytes prepared from control and K-*Arf6*-cKO mice were analyzed by western blotting with rabbit polyclonal anti-Arf6 antibody as previously reported^23^. Tubulin in keratinocytes was also detected using anti-tubulin antibody (T6199, Sigma-Aldrich) as an internal standard. Proteins reacted with the antibodies were visualized with Chemi-Lumi One (nacalai tesque), and signals were detected by the luminescent image analyzer LAS-4000 mini (Fujifilm).

### Statistical Analysis

All measurements describe mean ± SEM of at least three independent experiments. Statistical significance was determined using Student’s *t*-test and one-way ANOVA with Tukey’s HSD (honest significant difference), Scheffe’s post-hoc or Dunnett’s multiple comparison tests, and values of *P* < 0.05 were considered as statistically significant.

## Additional Information

**How to cite this article**: Miura, Y. *et al*. The small G protein Arf6 expressed in keratinocytes by HGF stimulation is a regulator for skin wound healing. *Sci. Rep.* **7**, 46649; doi: 10.1038/srep46649 (2017).

**Publisher's note:** Springer Nature remains neutral with regard to jurisdictional claims in published maps and institutional affiliations.

## Supplementary Material

Supplementary Information
