# Supplementary material for: The small G protein Arf6 expressed in keratinocytes by HGF stimulation is a regulator for skin wound healing
Source: Sci Rep. 2017 Apr 21;7:46649. doi: 10.1038/srep46649 (PMC5399375; doi:10.1038/srep46649)
Supplement: Supplementary Information [file srep46649-s1.pdf]

Supplementary Figure

The small G protein Arf6 expressed in keratinocytes by HGF stimulation is a regulator for skin wound healing

Yuki Miura, Ngo Thai Bich Van, Momoko Furuya, Hiroshi Hasegawa, Satoru Takahashi, Naohiro Katagiri, Tsunaki Hongu, Yuji Funakoshi, Norihiko Ohbayashi, Yasunori Kanaho

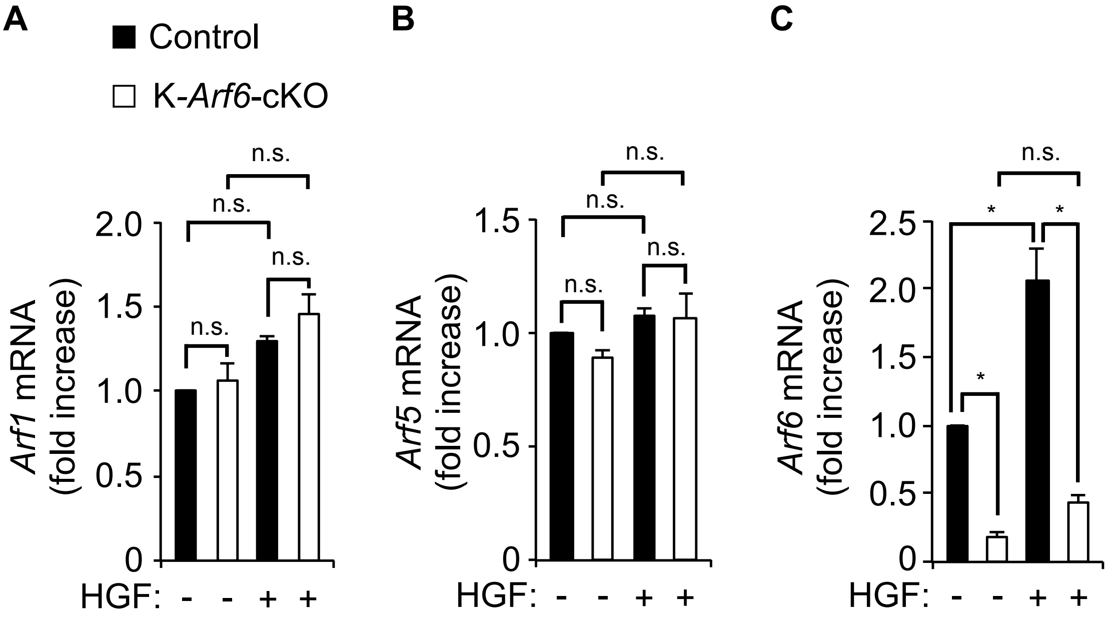

Supplementary Figure S1. Expression levels of *Arf1, 5* and *6* mRNA in primary cultured keratinocytes prepared from control and K-*Arf6*-cKO mice. Primary cultured keratinocytes prepared from control and K-*Arf6*-cKO mice were stimulated without or with 50 ng/ml of HGF for 3 hr, then *Arf1* (**A**), *Arf5* (**B**) and *Arf6* mRNA levels (**C**) were analyzed by qPCR. Data show the means ± SEM from three independent experiments. Statistical significance was assessed by one-way ANOVA with Scheffe's post-hoc test; $^{*}P<0.05$, n.s., not significant.
